# The M91 metallopeptidase domain of the T6SS effector protein SED_RS06335 encoded in *Salmonella* Dublin SPI-19 exhibits antibacterial activity

**DOI:** 10.1186/s13104-026-07931-2

**Published:** 2026-06-25

**Authors:** Carla Vargas-del Río, Ayleen Parra-Calisto, Carlos J. Blondel, Felipe Reyes-Méndez, Fernando A. Amaya, Carlos A. Santiviago, Andrea Avilés, Victoria Soriano-Mora, Daniel Chacín, David Pezoa

**Affiliations:** 1 Núcleo de Microbiología Traslacional para la Vigilancia e Innovación en Sistemas Sanitarios, Ambientales y Productivos MICRA, Facultad de Medicina Veterinaria y Agronomía, Universidad de Las Américas, Campus Providencia, Manuel Montt 948, Santiago, Chile; 2 Department of Chemical Engineering, Biotechnology and Materials, Centre for Biotechnology and Bioengineering (CeBiB), Universidad de Chile, Santiago, Chile; 3 Institute of Biomedical Sciences, Faculty of Medicine, Universidad Andres Bello, Santiago, Chile; 4 Laboratorio de Microbiología, Departamento de Bioquímica y Biología Molecular, Facultad de Ciencias Químicas y Farmacéuticas, Universidad de Chile, Santiago, Chile

**Keywords:** *Salmonella* Dublin, T6SS, SED_RS06335, M91 metallopeptidase domain, Antibacterial activity

## Abstract

**Objective:**

The Type VI Secretion System (T6SS) is a contractile apparatus made of several proteins playing a significant role in the fitness and virulence of many Gram-negative bacteria. The SPI-19 T6SS gene cluster is a virulence factor of *Salmonella* Dublin contributing to host colonization and antibacterial activity. Previously, we demonstrated that SED_RS06335 (an Rhs protein with a C-terminal M91 domain) is responsible for antibacterial activity of the T6SS_SPI−19_; however, the role of the M91 domain in this phenotype is unknown. Thus, the objective of this study is to provide experimental evidence that the antibacterial activity of SED_RS06335 effector is attributable to its C-terminal M91 domain.

**Results:**

Here, we determine through interbacterial competition and heterologous expression assays that M91 domain of SED_RS06335 displays antibacterial activity. Furthermore, a three-dimensional structural model of M91 domain revealed a high similarity with the metallopeptidase neurotoxin type A (BotA) from *Clostridium botulinum*. Interestingly, our results suggest that the M91 domain is responsible for the antibacterial activity of the SED_RS06335 effector in *Salmonella* Dublin.

**Supplementary Information:**

The online version contains supplementary material available at https://doi.org/10.1186/s13104-026-07931-2.

## Introduction

**Fig. 1:**
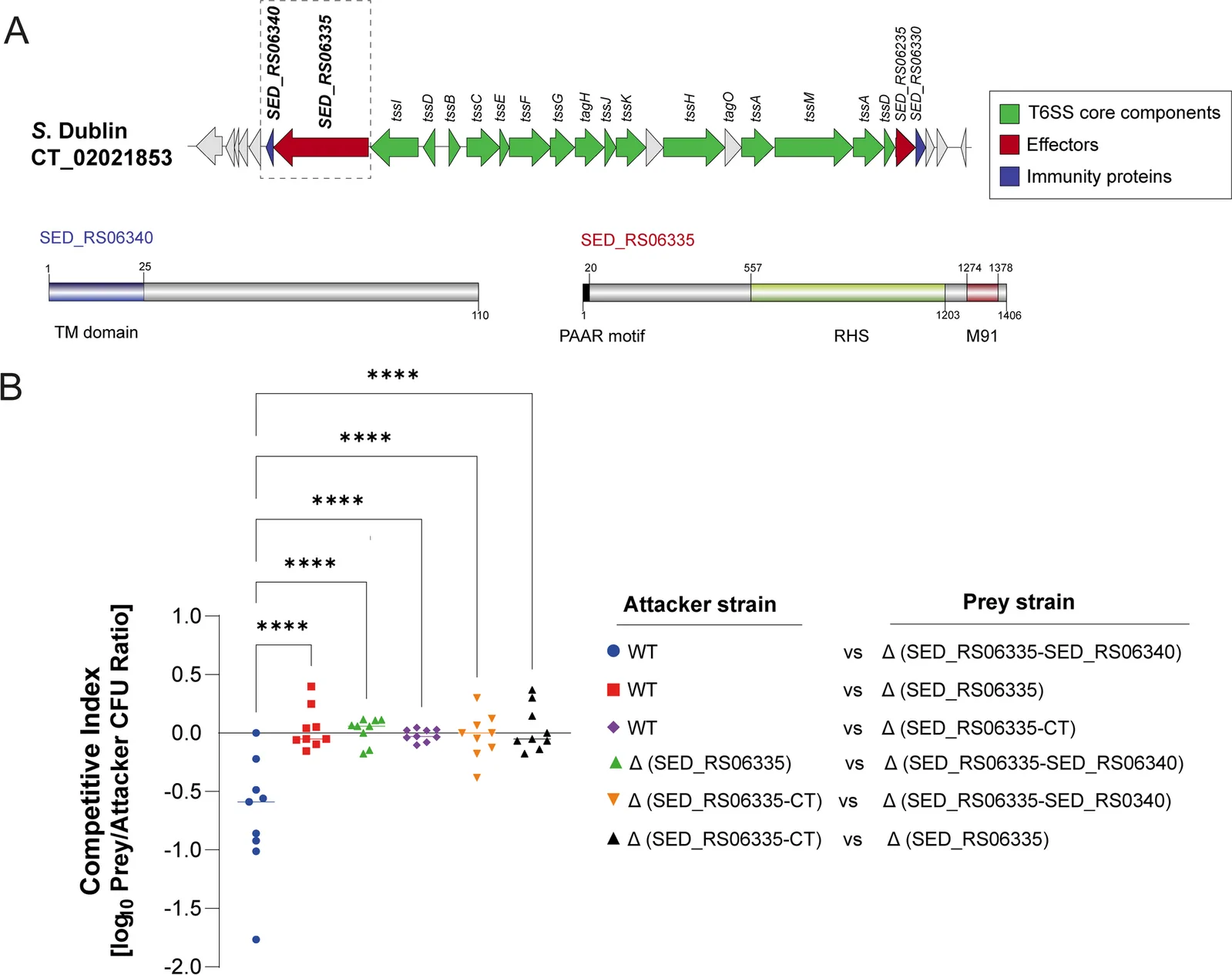
Contribution of the C-terminal M91 metallopeptidase domain of SED_RS06335 to antibacterial activity in*S*. Dublin CT_02021853. **A** Schematic representation of the *S*. Dublin CT_02021853 SPI-19 T6SS gene cluster and the C-terminal M91 metallopeptidase domain of SED_RS06335. The figure illustrates the genetic organization of the SPI-19 T6SS gene cluster in *S*. Dublin CT_02021853, highlighting the specific location of the *SED_RS06335* / *SED_RS06340* E/I module. *SED_RS06335* is shown in red, while *SED_RS06340* (encoding its putative cognate immunity protein) is shown in blue. ORFs encoding T6SS core components are shown in green. **B** Interbacterial competition assays. Bacterial cultures of attacker and prey strains were adjusted to an OD_600nm_ of 0.5 and then mixed at a 1:1 ratio. Each mixture was plated on MacConkey agar plates and incubated at 37 °C for 24 h in triplicate. Colony-forming units (CFU) were calculated by counting the colonies that grown after plating 10-fold serial dilutions on LB agar containing the respective antibiotics. The data shows prey-to-attacker CFU ratio expressed as log10 and normalized to the inoculum. Statistical significance was calculated by a one-way ANOVA test and Tukey’s multiple comparisons using GraphPad Prism 9.0 software (****p < 0.0001)

The Type VI Secretion System (T6SS) has been defined as a multiprotein nanomachine comprising 13 structural components and a variable number of accessory proteins [1, 2], delivering versatile effector proteins into prokaryotic and/or eukaryotic cells [3, 4]. Thus, T6SS has emerged as a significant fitness and virulence factor for numerous Gram-negative bacteria [5–8]. Recent studies have elucidated the significance of T6SSs for *Salmonella* pathogenesis [9–18]. *Salmonella* possesses five T6SS gene clusters, which are encoded in pathogenicity islands SPI-6, SPI-19, SPI-20, SPI-21, and SPI-22 that belong to different evolutionary lineages [19, 20]. In *S.* Dublin, which is a cattle-adapted pathogen, the T6SS_SPI-19_ has been shown to contribute to intestinal colonization, antibacterial activity and cytotoxicity against macrophages [21, 22]. In a previous report, we demonstrated that the *SED_RS06335*/*SED_RS06340* Effector/Immunity protein module contributes to interbacterial competition in *S*. Dublin [18]. The candidate T6SS effector SED_RS06335 is a 1,406 amino acid Rhs protein encoding a PAAR motif in its N-terminal domain and a C-terminal M91 domain with predicted metallopeptidase activity (Fig. 1A). Of note, the role played by the C-terminal M91 domain in the antibacterial activity of SED_RS06335 remains to be elucidated. In this work, we provide the first experimental evidence indicating that the M91 metallopeptidase domain is required for the antibacterial activity of SED_RS06335 by means of interbacterial competition and heterologous expression assays. Furthermore, a three-dimensional structural model of the C-terminal domain of SED_RS06335 (residues 1,274 to 1,378) revealed a high similarity between the predicted structure and the M91 metallopeptidase domain of botulinum neurotoxin type A (BotA) from *Clostridium botulinum*.

## Materials and methods

### Bacterial strains and growth conditions

The bacterial strains utilized in this study are enumerated in Table 1. Bacteria were cultivated in Luria-Bertani (LB) broth (10 g/L tryptone, 5 g/L yeast extract, 5 g/L NaCl) at 37 °C with aeration. LB broth was augmented with ampicillin (Amp; 100 µg/mL), kanamycin (Kan; 50 µg/mL), or chloramphenicol (Cm; 20 µg/mL), as required. To solidify LB medium, agar (15 g/L) was added. In the context of interbacterial competition assays, bacterial cultures were cultivated on McConkey agar plates at a temperature of 37 °C for a duration of 24 h. For heterologous expression assays, bacteria were incubated at 37 °C for 24 h on LB agar plates supplemented with Kan, Cam, and the corresponding inducers (i.e., 0.1 mM IPTG and/or 0.4% L-arabinose).

**Table 1:** Bacterial strains and plasmids utilized in this work

Strains	Features	Source or references
*Escherichia coli*		
DH5α	F^−^ Φ80∆*lacZ*(M15) ∆(*lacZYA-argF*)*U169* deoR *recA1 endA1 hsdR17*(r_k_^−^, m_k_^+^) *phoA supE44 thi-1 gyrA96 relA1* λ^−^	Laboratory collection
BL21	F^–^ *ompT gal dcm lon hsdS*_*B*_*(r*_*B*_^*–*^*m*_*B*_^*–*^*) λ(DE3 [lacI lacUV5-T7p07 ind1 sam7 nin5]) [malB*^*+*^*]*_*K−12*_*(λ*^*S*^*)*	Laboratory collection
*Salmonella* Dublin		
CT_02021853	Wild-type strain	Laboratory collection
Δ*SED_RS06335*::FRT Δ*phoN*::Kan	CT_02021853 Δ*SED_RS06335*::FRT Δ*SED_RS22655*::Kan	[18]
Δ*SED_RS06335*::FRT Δ*phoN*::Cam	CT_02021853 Δ*SED_RS06335*::FRT Δ*SED_RS2265*)::Cam	[18]
Δ(*SED_RS06335*-*SED_RS06340*)::FRT Δ*phoN*::Cam	CT_02021853 Δ(*SED_RS06335*-*SED_RS06340*)::FRT Δ*SED_RS22655*::Cam	[18]
Δ*SED_RS06335_CT*::Kan	CT_02021853 Δ*SED_RS06335*::Kan	This study
Δ*SED_RS06335_CT*::Cam	CT_02021853 Δ*SED_RS06335*::Cam	This study
Plasmids		
pKD46	*bla P*_*BAD*_ *bet gam exo oriR101*(TS), Amp^R^	[23]
pCP20	*bla cat cI857* λP_R_ *flp oriR101*(TS), Cam^R^, Amp^R^	[32]
pCLF2	Red-swap redesigned vector, Amp^R^, Cam^R^	GenBank HM047089
pCLF4	Red-swap redesigned vector, Amp^R^, Kan^R^	GenBank EU629214
pRSFDuet-1	*E. coli* expression vector. Kanamycin resistance. IPTG-inducible recombinant protein expression vector. The vector contains two multiple cloning sites (MCS1 and MCS2), each of which is preceded by a T7 promoter/*lac* operator and a ribosome binding site. RSF replicon	Novagen
pBAD33.1	*E. coli* expression vector. Arabinose-inducible recombinant protein expression vector. Ampicillin resistance. P15A replicon	[33]
pET20b	IPTG-inducible recombinant protein expression vector. Ampicillin resistance. This vector adds an N-terminal PelB signal sequence for expression of proteins in the periplasm. ColE1 replicon	Novagen
pET20b_SP_CT06335	pET20b derivative. Expressing the C-terminal domain of SED_RS06335 from *S*. Dublin CT_02021853 with a PelB signal sequence at the N-terminal and a His6 tag at the C-terminal.	This study
pRSFDuet-1_SP_CT06335	pRSFDuet-1 derivative. Expressing the C-terminal domain of SED_RS06335 from *S*. Dublin CT_02021853 with a PelB signal sequence at the N-terminal and a His6 tag at the C-terminal.	This study
pRSFDuet-1_CT06335	pRSFDuet-1 derivative. Expressing the C-terminal domain of SED_RS06335 from *S*. Dublin CT_02021853 with a His6 tag at the C-terminal	
pBAD33.1_TM_06340	pBAD33.1 derivative. Expressing the full-length *SED_RS06340* from *S*. Dublin CT_02021853 with a His6 tag at the C-terminal.	This study

### Standard DNA techniques

Plasmids were obtained by the “QIAprep Spin Miniprep Kit” (QIAGEN, MD, USA). PCR amplicons were extracted by means of the “QIAquick PCR Purification Kit” (QIAGEN, MD, USA). Analysis of DNA samples was performed in 1% agarose gels electrophoresis. To visualize DNA under UV light, RedGel (Biotium, CA, USA) was added. Oligonucleotides were designed based on the genome sequence of *S.* Dublin CT_02021853 (GenBank CP001144.1) using the SnapGene software (www.snapgene.com) and are listed in Table 2.

**Table 2:** Oligonucleotides used in this work

Primers	Sequence^a^
Mutagenesis	
SED_RS06335_H1 + P1	AGAAATAAAGATGAGCGGAAAACCAGCGGCGCGTCAGGGCGTGTAGGCTGGAGCTGCTTC
SED_RS06335_H2 + P2	ATCTTTATCATCAGTATTTCATCCTTGGTGGGATTCCCAT*CATATGAATATCCTCCTTAG*
SED_RS06340_H2 + P2	CTATGAAATATTAGTGATTATCTTCATATATATATATTCT*CATATGAATATCCTCCTTAG*
SED_RS06335_Out5	GCGGTATTTTTCTGAACGGCA
CT06335_H1 + P1	GAACCCTGTCACAGGTACAGATCCATTGGGATTAAAGTGA*GTGTAGGCTGGAGCTGCTTC*
CT06335_H2 + P2	TCTTTATCATCAGTATTTCATCCTTGGTGGGATTCCCATT*CATATGAATATCCTCCTTAG*
CT_06335_OUT3	TAATGATTTTTTGAAGATGC
phoN_SDu_(H1 + P1)	GTGAGTCTTTATGAAAAGTCGTTATTTAGTATTTTTTCTA*GTGCAGGCTGGAGCTGCTTC*
phoN_SDu_(H2 + P2)	ACTTTCACCTTCAGTAATTAAGTTCGGGGTGATCTTCTTT*CATATGAATATCCTCCTTAG*
phoN_SDu_Out5	TTGCCTGATCCGGAGTGA
K1	CAGTCATAGCCGAATAGCCT
C3	CAGCTGAACGGTCTGGTTATAGG
Cloning	
pET20b_NcoI_SP_06335_F	CACACCATGGGTGGTTCTGGTGTGACTTTTAAGGGTGACGA
pET20b_XhoI_SP_06335_R	CACACTCGAGATGTCCTGGTCCGTCGTCAT
pET20b_OUT5_F	GGGAGACCACAACGGTTTCC
pET20b_OUT3_R	CCTTTCGGGCTTTGTTAGCA
pRSFDuet1_NdeI_woSP_06335_F	CACACATATGGTGACTTTTAAGGGTGACGA
pRSDDuet1_XhoI_wSP_06335_R	CACACTCGAGATGTCCTGGTCCGTCGTCAT
DuetUP2 Primer	TTGTACACGGCCGCATAATC
T7 Terminator Primer	GCTAGTTATTGCTCAGCGG
pBAD33.1_NdeI_06340_TM_F	CACACATATGATAAAGATAAGCCCTCCCTG
pBAD33.1_HindIII_06340_TM_R	CACAAAGCTTTTAGTGGTGATGGTGATGATGGTGATTATCTTCATATATAT
pBAD Forward	ATGCCATAGCATTTTTATCC
pBAD Reverse	GATTTAATCTGTATCAGG

^a^Underline indicates the region that anneals to the 5′ or 3′ end of the antibiotic resistance cassette used for the mutagenesis

PCR reactions were performed in mixes that contain 1X buffer, 2 mM MgCl_2_, 100 nM dNTPs, 100 nM of each forward and reverse oligonucleotides, 100 ng of template DNA, and 0.5 to 1 U of Phusion High-Fidelity DNA Polymerase (NEB, USA). DNA amplifications were performed using standard conditions: 1 min at 98 °C, 30–35 cycles of 98 °C for 10 s, 55 °C for 30 s, and 72 °C for 15–30 s/kb, followed by a final extension at 72 °C for 10 min. All amplifications were conducted in a “MultiGeneTC9600-G” thermal cycler (LabNet, NJ, USA).

### Interbacterial competition assays

To this end, we constructed mutant strains of the SED_RS06335-encoding gene, the *SED_RS06335* / *SED_RS06340* E/I module, or the portion of *SED_RS06335* encoding the C-terminal domain of the corresponding effector by the one-step inactivation procedure using the Lambda Red recombination system (Table 1) [23], with modifications [24]. Interbacterial competition experiments were conducted using different combinations of attacker and prey strains, as described by ref. [14]. Briefly, 25µL aliquots of an overnight mix composed of attacker and prey strains (1:1) were dispensed as spots in triplicates onto McConkey agar plates, a condition that can induce T6SS gene expression in *Salmonella* [22]. Then, the bacterial spots were recovered from the McConkey agar plates and colony-forming units (CFU) were calculated by counting the colonies that grown after plating serial dilutions on LB agar containing the respective antibiotics. The ratio of prey to attacker was standardized to the inoculum ratio and expressed as log10. Three biological replicates were performed. Statistical significance was calculated by a one-way ANOVA test and Tukey’s multiple comparisons using GraphPad Prism 9.0 software.

### Overexpression of effector/immunity pair in *E. coli*

*E. coli* BL21(DE3) strains harboring either empty control plasmids (pRSFDuet-1 and pBAD33.1) or recombinant pRSFDuet-1 constructs expressing SED_RS06335-CT, with or without the N-terminal PelB signal peptide, in combination with pBAD33.1 expressing SED_RS06340, were grown overnight at 37 °C in LB medium supplemented with 50 µg/mL kanamycin, 20 µg/mL chloramphenicol, and 0.4% glucose. Overnight cultures were subsequently diluted 1:100 into fresh LB medium containing the appropriate antibiotics for plasmid maintenance and grown to an OD₆₀₀ of 0.1. Cultures were then incubated for an additional 1 h at 37 °C, after which OD₆₀₀ was recorded. Protein expression was induced by the addition of 0.02 mM IPTG and 0.4% L-arabinose, followed by incubation at 37 °C with shaking at 180 rpm for 7 h. Optical density at 600 nm (OD₆₀₀) was measured at hourly intervals.

### Heterologous toxicity assays

To evaluate if expression of the M91 metallopeptidase domain is toxic to *E. coli* BL21, a DNA fragment encoding SED_RS06335-CT with (SP-SED_RS06335-CT) or without (SED_RS06335-CT) an N-terminal PelB signal peptide was cloned into the expression vector pRSFDuet-1 (Table 1). Furthermore, the DNA fragment that encodes the putative SED_RS06340 immunity protein was cloned into the pBAD33.1 vector (Table 1). This construction allows SED_RS06340 expression under the control of the PBAD promoter, that can be induced by L-arabinose and repressed by D-glucose. *E. coli* BL21 harboring empty control plasmids, or plasmids expressing SED_RS06335-CT with or without the N-terminal PelB signal peptide and/or SED_RS06340, were serially diluted and plated on LB agar containing 0.02 mM IPTG and 0.4% L-arabinose.

### Protein structure prediction and homology modelling

Protein structure models of the C-terminal domain of SED_RS06335 in complex with Zn^+ 2^ as a ligand, were obtained using the protein structure prediction software Alphafold3 (Alphafold Server) [25] with default parameters. The model that demonstrated the most optimal predicted template modeling (pTM) score and interface predicted template modeling (ipTM) score was selected for subsequent analyses. Protein structures similar to the modelled SED_RS06335-CT were identified by Foldseek [26]. Protein structure visualization, template alignment, and superposition of structures were performed using Pymol v3.1 and the Matchmaker tool of UCSF ChimeraX [27].

## Results and discussion

### The C-terminal domain of SED_RS06335 exhibits antibacterial activity

First, to test the role played by the C-terminal M91 domain in the antibacterial activity of SED_RS06335 we performed interbacterial competition experiments using different combinations of attacker and prey strains. Our results showed that the mutant with the entire E/I module (*SED_RS06335* / *SED_RS06340*) deleted, exhibited a diminished survival rate following co-incubation with the wild-type strain (Fig. 1B).

**Fig. 2:**
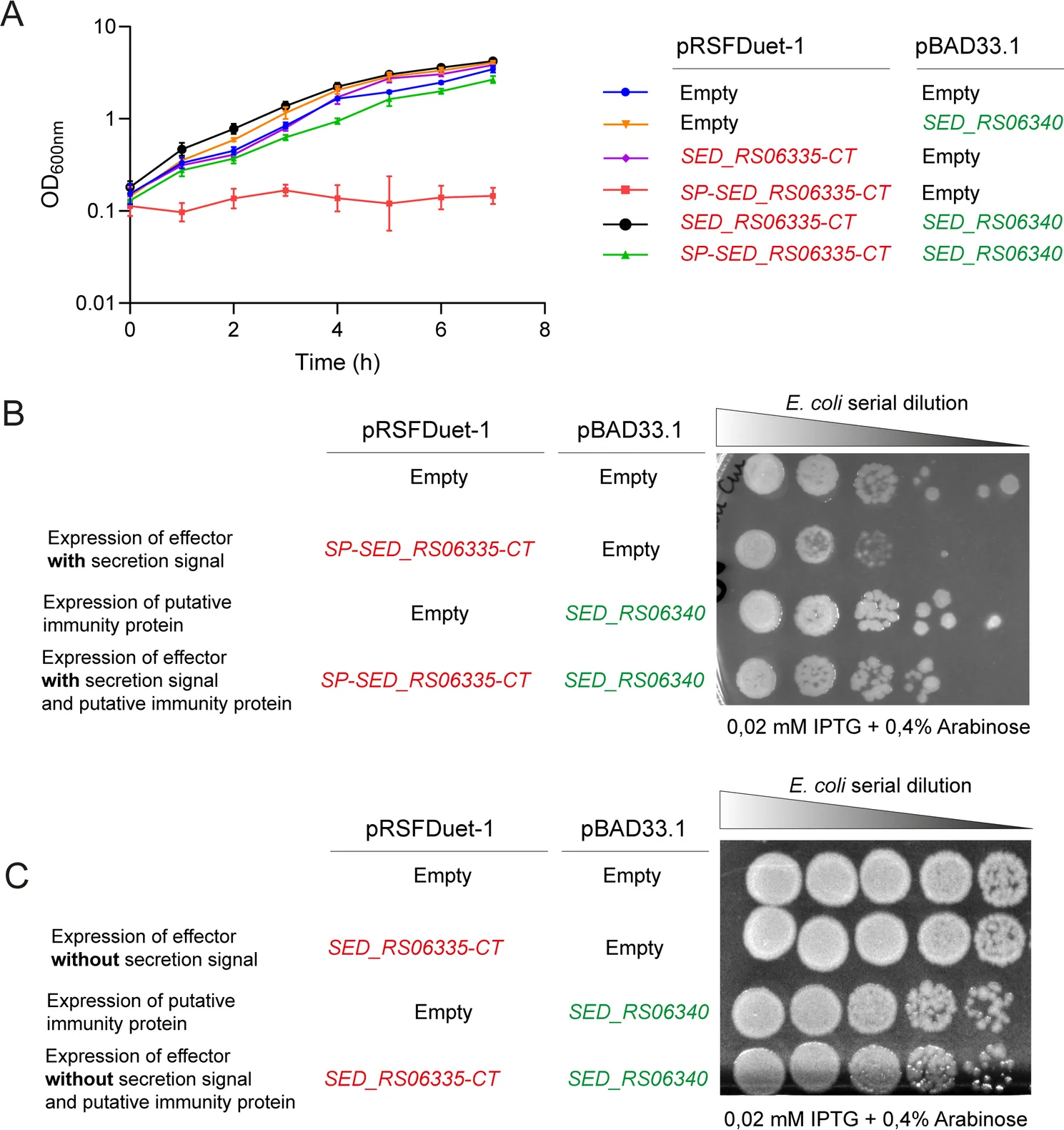
Heterologous expression assays. Bacterial growth curves in liquid media (**A**) and agar plate assays (**B** and **C**) of strains BL21 harboring empty vector controls (pRSFDuet-1, pBAD33.1) or plasmids expressing the C-terminal region of SED_RS06335 with an N-terminal PelB signal peptide (SP-SED_RS06335-CT) and/or the SED_RS06340 immunity protein or the C-terminal region of SED_RS06335 without the signal peptide (SED_RS06335-CT) and/or the SED_RS06340 immunity protein. For liquid cultures bacteria O/N cultures were diluted in 1/100 in the presence/absence of 0.02 mM IPTG and/or 0.4% L-arabinose to induce the effector and the putative immunity protein, respectively and bacterial growth analyzed by measuring OD600nm For growth on agar plates, bacteria were diluted in LB broth (1:4) and aliquots (5 µL) were plated in spots onto LB agar plates supplemented with kanamycin and chloramphenicol plus 0.02 mM IPTG and/or 0.4% L-arabinose to induce the effector and the putative immunity protein, respectively. Finally, bacteria were grown at 37 °C for 24 h

This was expected, since this mutant lacks the cognate immunity protein gene (*SED_RS06340*) of SED_RS06335. In contrast, mutant strains lacking the SED_RS06335-encoding gene or its C-terminal region that retained the SED_RS06340 immunity protein, did not exhibit a diminished survival rate following co-incubation with the wild-type strain (Fig. 1B); this is explained because both strain keeps intact the immunity protein gene and thus, counteracts the toxic activity of the SED_RS06335 effector delivered by the attacker strain (wild-type). In contrast, the survival of the mutant lacking the complete SED_RS06335/SED_RS06340 module was not affected by an attacker strain which lacks the respective effector gene (∆SED_RS06335), as expected. Interestingly, the survival of prey mutants that lack either the *SED_RS06335* gene (and keeping intact the immunity protein encoding gene SED_RS06340) or the *SED_RS06335* / *SED_RS06340* E/I module (strain that do not have the SED_RS06340 immunity protein gene) had a very similar survival rate following co-incubation with the attacker mutant strain lacking the C-terminal region of SED_RS06335 (Fig. 1B). This observation is explained because the attacker strain (∆SED_RS0335-CT) lost its capacity to kill the prey bacteria since it does not posses the M91 toxin domain. These results demonstrate that the predicted C-terminal M91 metallopeptidase domain of SED_RS06335, but not the N-terminal PAAR motif nor the Rhs domain, is responsible for the antibacterial activity of this effector protein. The latter was expected as PAAR motifs are key players in the assembly and firing mechanism of a T6SS [28, 29]. Then, to gain insights into the mechanism of action of SED_RS06335, heterologous toxicity assays were performed. First, growth curve analyses of *E. coli* BL21(DE3) strains harboring either empty control plasmids (pRSFDuet-1 and pBAD33.1) or recombinant pRSFDuet-1 constructs expressing SED_RS06335-CT, with or without the N-terminal PelB signal peptide, in combination with pBAD33.1 expressing SED_RS06340 were performed (Fig. 2A). This signal peptide was incorporated since the putative immunity protein SED_RS06340 harbor a transmembrane domain that is predicted to localize this protein in the periplasmic space. Periplasmic expression of SP-SED_RS06335-CT in *E. coli* following IPTG induction led to a complete arrest of bacterial growth within 1 h post-induction (Fig. 2A). Co-expression of SP-SED_RS06335-CT with the predicted cognate immunity protein, SED_RS_06340, restored bacterial growth, whereas cytoplasmic expression of SED_RS06335-CT alone had no detectable impact on *E. coli* growth (Fig. 2A). Collectively, these findings indicate that SED_RS06335 exerts its toxic activity in the periplasm and that SED_RS06340 functions as its cognate immunity protein, likely through its transmembrane domain that enables periplasmic localization and neutralization of effector-mediated toxicity.

Then, heterologous expression assays were conducted on agar plates. As shown in Fig. 2B, expression of SP-SED_RS06335-CT was mildly toxic to *E. coli*, while no toxicity was observed when only the SED_RS06340 immunity protein was expressed. The expression conditions (0.02mM IPTG and 0.4% L-arabinose) allowed high protein production of both constructs (Figure S1).

As expected, the toxicity of SP-SED_RS06335-CT was alleviated upon co-expression of the putative immunity protein SED_RS06340 (Fig. 2B). Notably, when SED_RS06335-CT was expressed in the cytosol no growth inhibition was observed (Fig. 2C), further supporting the notion that SED_RS06335 corresponds to a T6SS effector protein targeted to the periplasm. Other putative T6SS effector proteins, such as Tpe2 in *P. aeruginosa* [30] and Ec154_02824 in *E. coli* (STEC) O22:H8 [31], have been identified to harbor M91 domains associated with C-terminal extensions of Rhs effectors in bioinformatic analyses performed in *E. coli* [29] and shiga-toxin producing *E. coli* [31]. However, no further functional or biochemical characterization of this M91 domain has been performed yet.

### The C-terminal M91 peptidase domain of SED_RS06335 has predicted structural similarity to the catalytic domain of toxins from *Clostridium botulinum*

**Fig. 3:**
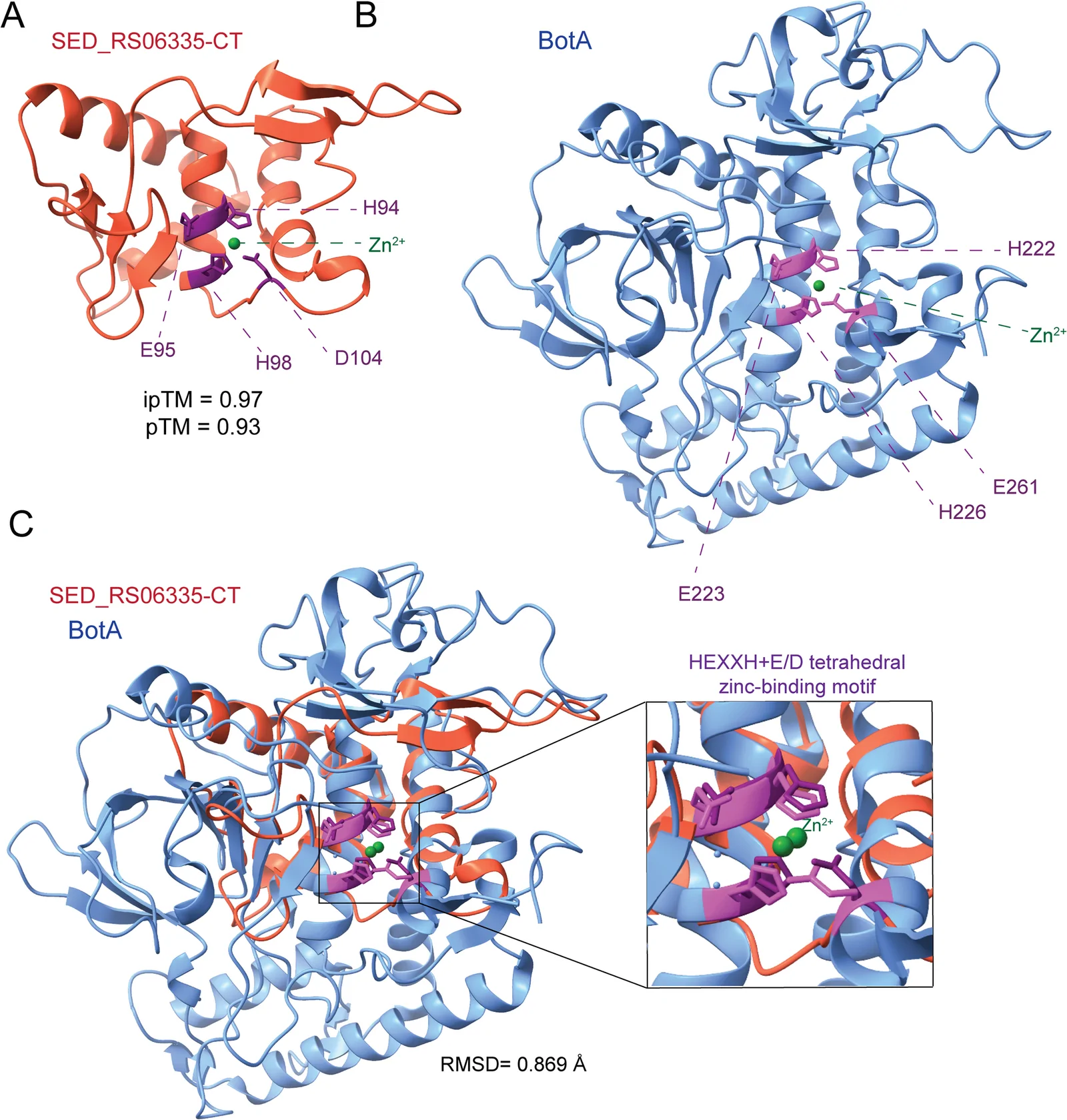
Comparative homology model of the C-terminal domain of SED_RS06335 superimposed on the known structure of the catalytic domain of BotA of*C. botulinum*. **A** Alphafold3 model of the C-terminal region of SED_RS06335 (residues 1,274 to 1,378) in complex with Zn^2+^. The amino acids of the predicted catalytic site in complex with the zinc ion are shown in purple. **B** Crystal structure of the catalytic domain of BotA (PDB 2G7Q). Amino acids of the catalytic site (highlighted in purple) are shown in complex with the zinc ion (highlighted in green). **C** Superimposition of the Alphafold3 structure of the C-terminal region of SED_RS06335 and BotA. The catalytic triads (HEXXH + E/D) of both proteins are highlighted in the black box

Finally, to gain further insight into the predicted biochemical activity of SED_RS06335, a 3D structural modelling of the C-terminal domain of SED_RS06335 (residues 1,274 to 1,378) was constructed by Alphafold3. As shown in Fig. 3A and S2, Alphafold3 allowed us to model the C-terminal domain of SED_RS06335 in complex with Zn^2+^ (with high confidence ipTM and pTm scores of 0.97 and 0.91, respectively) coordinated by residues H1367, E1368, H1371 and D1377 present in the HEXXH + E/D motif. Foldseek analysis identified that the BotA neurotoxin of *C. botulinum* (PDB 2G7Q) (Fig. 3B) has a similar structure to the model of SED_RS06335-CT. As shown in Fig. 3C, there is high similarity between the predicted structure of the M91 metallopeptidase domain in SED_RS06335-CT with the catalytic domain of BotA, sharing the same catalytic pocket (Fig. 3C).

## Strenghts and limitations

We determined that the predicted C-terminal M91 domain of SED_RS06335 encoded in the SPI-19 T6SS gene cluster of *S*. Dublin is required for antibacterial activity during interbacterial competition assays and is toxic when expressed in the periplasm of *E. coli*. Interestingly, this is the first demonstration of the antibacterial activity of M91 domain associated with a T6SS effector. Despite the high similarity between the predicted structure of the M91 metallopeptidase domain in SED_RS06335-CT with the catalytic domain of BotA, it is important to note that this model has not been verified, and the nature of the cofactor remains to be determined. Further work is required to determine the molecular mechanisms behind the toxic effect of the M91 domain, this will allow us to gain further insights on the role of T6SSs to the fitness and pathogenic potential of different *Salmonella* serotypes.

## Conclusions

We provide the first demonstration that the M91 metallopeptidase domain is responsible for the antibacterial activity of SED_RS06335, as evidenced by interbacterial competition and heterologous expression assays. In addition, a structural model of the C-terminal domain of SED_RS06335 revealed a high similarity between the predicted structure and the M91 metallopeptidase domain of botulinum neurotoxin type A (BotA) from *Clostridium botulinum*. This supports the notion that the predicted M91 metallopeptidase domain of SED_RS06335 protein is functional and contributes to the antibacterial activity of this effector protein in *S*. Dublin CT_02021853.

## Supplementary Information

Below is the link to the electronic supplementary material.

Supplementary Material 1.

Supplementary Material 2.

## Data Availability

The datasets gathered by the corresponding author during the current study are available upon reasonable request.
