# Supplementary material for: The M91 metallopeptidase domain of the T6SS effector protein SED_RS06335 encoded in Salmonella Dublin SPI-19 exhibits antibacterial activity
Source: BMC Res Notes. 2026 Jun 25;19:347. doi: 10.1186/s13104-026-07931-2 (PMC13556075; doi:10.1186/s13104-026-07931-2)
Supplement: Supplementary file 1 [file 13104_2026_7931_MOESM1_ESM.pdf]

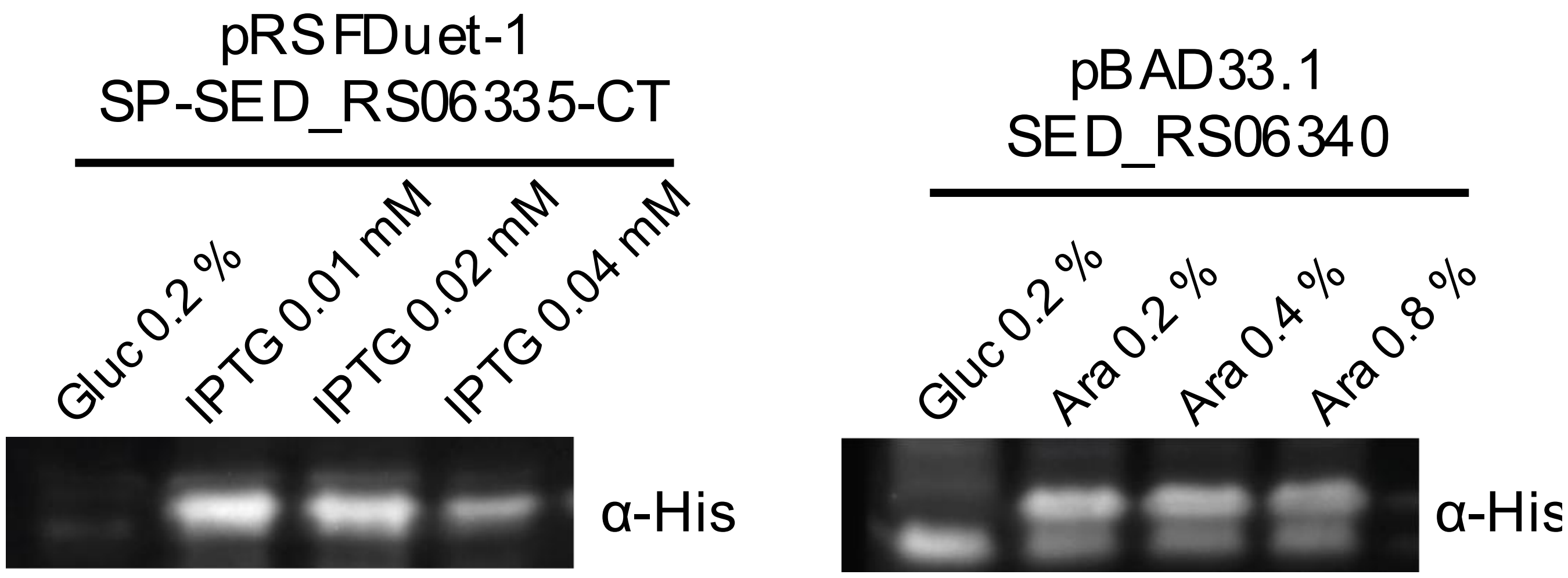
pRSFDuet-1
SP-SED_RS06335-CT
Gluc 0.2 %
IPTG 0.01 mM
IPTG 0.02 mM
IPTG 0.04 mM
α-His
pBAD33.1
SED_RS06340
Gluc 0.2 %
Ara 0.2 %
Ara 0.4 %
Ara 0.8 %
α-His
