# Supplementary material for: The M91 metallopeptidase domain of the T6SS effector protein SED_RS06335 encoded in Salmonella Dublin SPI-19 exhibits antibacterial activity
Source: BMC Res Notes. 2026 Jun 25;19:347. doi: 10.1186/s13104-026-07931-2 (PMC13556075; doi:10.1186/s13104-026-07931-2)

AF3 model of SED_RS06335-CT in complex with Zinc ion

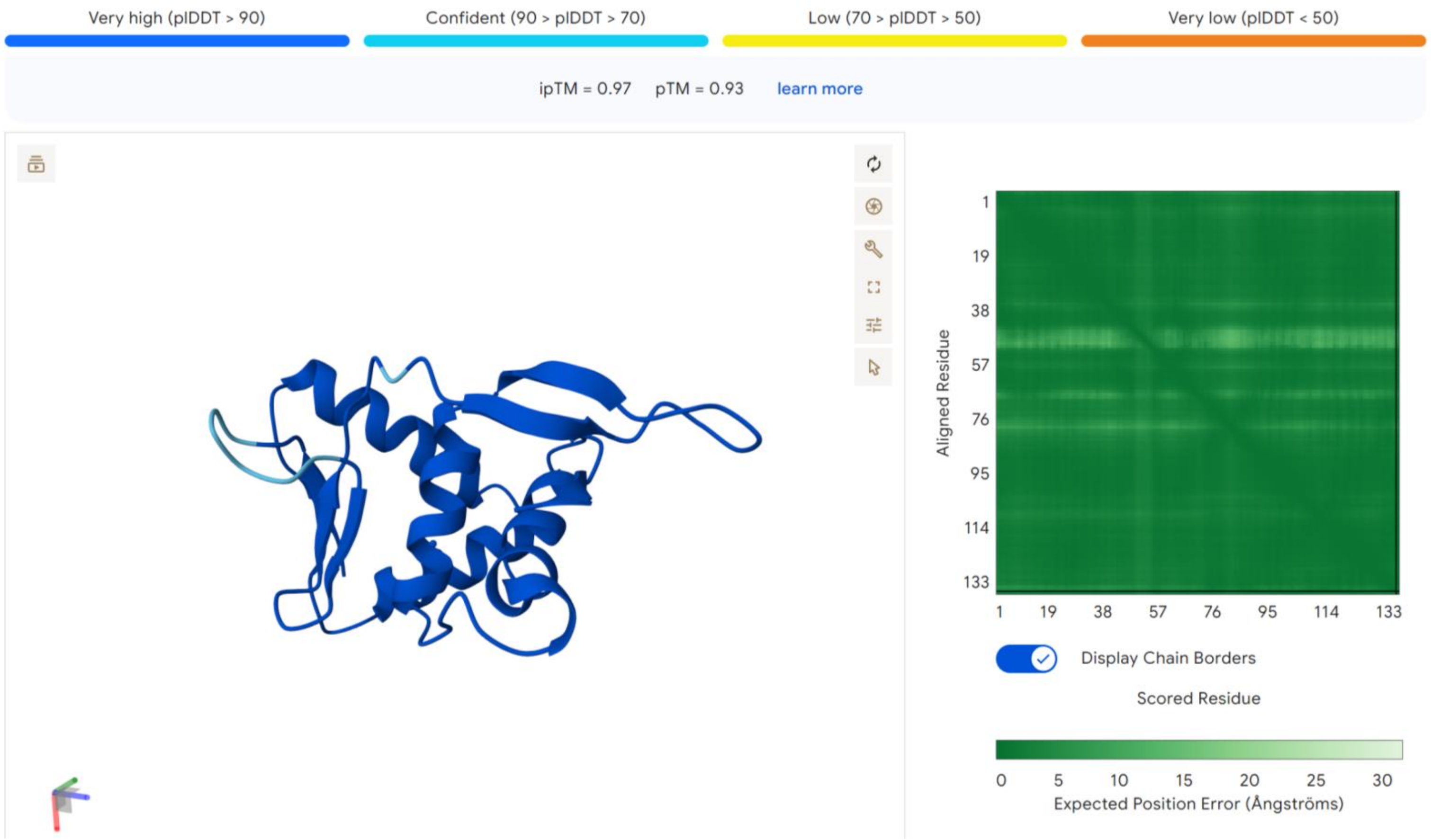

Supplement: Supplementary file 2 [file 13104_2026_7931_MOESM2_ESM.pdf]
